# Health and functional advantages of cheese containing soy protein and soybean-derived casein

**DOI:** 10.3389/fpls.2024.1407506

**Published:** 2024-07-23

**Authors:** Mark Messina, Virginia Messina

**Affiliations:** ^1^ Nutrition Science and Research, Soy Nutrition Institute Global, Jefferson, MO, United States; ^2^ Nutrition Matters, Inc., Pittsfield, MA, United States

**Keywords:** soybeans, cheese, soy protein, casein, animal-free, functional, orosensory, nutrition

## Abstract

The global food system faces a challenge of sustainably producing enough food, and especially protein, to meet the needs of a growing global population. In developed countries, approximately 2/3 of protein comes from animal sources and 1/3 from plants. For an assortment of reasons, dietary recommendations call for populations in these countries to replace some of their animal protein with plant protein. Because it is difficult to substantially change dietary habits, increasing plant protein may require the creation of novel foods that meet the nutritional, orosensory, and functional attributes consumers desire. In contrast to plant-based milks, plant-based cheeses have not been widely embraced by consumers. The existing plant-based cheeses do not satisfactorily mimic dairy cheese as plant proteins are unable to replicate the functional properties of casein, which plays such a key role in cheese. One possible solution to overcome current constraints that is currently being explored, is to produce hybrid products containing soy protein and soybean-derived casein. Producing soybean-derived casein is possible by utilizing traditional genetic engineering tools, like *Agrobacterium*-mediated plant transformation, to express genes in soybeans that produce casein. If a cheese containing soy protein and soybean-derived casein satisfactorily mimics dairy, it presents an opportunity for increasing plant protein intake since US dairy cheese consumption has been steadily increasing. Soybeans are an excellent choice of crop for producing casein because soybeans are widely available and play a large role in the US and world food supply. Additionally, because a casein-producing soybean offers soybean farmers the opportunity to grow a value-added crop, expectations are that it will be welcomed by the agricultural community. Thus, there are benefits to both the consumer and farmer.

## Introduction

The global food system faces a major challenge of sustainably producing sufficient food, and especially protein, for a global population expected to reach nearly 10 people by 2050. Sustainable food patterns are those that are nutritionally adequate, economically affordable, socially acceptable, and compatible with goals to stem climate change (Auestad and Fulgoni, 2015). By one estimate, as much as 80% more protein needs to be produced by the year 2050 to meet global demand (Henchion et al., 2017).

Developing countries derive approximately 2/3 of protein from animal sources and 1/3 from plants (Halkjaer et al., 2009; Salome et al., 2020; Hoy et al., 2022). For an assortment of reasons, but especially because of the postulated health benefits, authorities call for populations in developed countries to consume more plant protein (Willett et al., 2019). In support of these recommendations are numerous epidemiologic studies that have found plant-based diets are associated with reduced chronic disease risks. For example, among the 126,394 participants of the UK Biobank, adherence to a healthful plant-based diet was associated with lower risks of total mortality, cancer and cardiovascular disease (Thompson et al., 2023). In the US, long-term follow up of women in the Nurses’ Health Study I (n=69,949) and II (n=90,239) and men in the Health Professionals Follow-Up Study (n=40,539), revealed that a healthy plant-based diet was associated with a reduced risk of developing type 2 diabetes (T2D) (Satija et al., 2016). Finally, a systematic review and meta-analysis of observational studies involving 2,230,443 participants with 60,718 cases of incident T2D, 157,335 CVD cases, 57,759 cancer cases, and 174,435 deaths, found that higher adherence to a healthy plant-based dietary pattern was associated with lower risks of each of those diseases (Wang et al., 2023).

However, it is well established that consumers have a difficult time substantially changing their diet. For example, in the United States, the recommendation to consume no more than 10% of calories as saturated fat has never been met (Honors et al., 2014), and Americans consume only about half the recommended fiber intake and markedly lower amounts of whole grains, and fruits and vegetables than recommended (Tao et al., 2022). To effect dietary change, new practices should not diverge too much from consumers’ usual behavior (Ryan and Deci, 2000).

Therefore, for plant protein intake to increase, and to achieve a dietary animal to plant protein of 1:1, as some have recommended for developed countries, novel foods may need to be created (Grasso et al., 2021). These foods need to allow individuals to consume more plant protein without requiring them to compromise on taste, nutrition, or functionality. Developing foods that combine plant and animal protein is one possible solution.

Although such hybrid products, such as ground beef combined with soy protein (Thrane et al., 2017; Grasso et al., 2019; Bakhsh et al., 2021), and cow’s milk combined with plant milks (Abdulqader et al., 2022; Wan et al., 2022) are available, they have not yet been accepted on a wide scale. However, the results of a recent Swiss survey provide reason for optimism about their increased acceptance. Of the 1,000 respondents, 2/3 consume animal foods and plant-based substitute products at the same meal (Finkel, 2024). This observation suggests people may be willing to try hybrid meats.

One novel approach to producing hybrid products that is currently being explored is to combine soy protein with soybean-derived casein, the primary protein in cow’s milk (Daniloski et al., 2021). Producing soybean-derived casein is possible by utilizing traditional genetic engineering tools, like *Agrobacterium*-mediated plant transformation, to express this protein in plants, also known as “molecular farming“ (Gelvin, 2003; Hwang et al., 2017) or plant-grown proteins.

Soybeans are a crop of immense importance to the world’s food supply, both in the form of animal feed and as food directly consumed by humans (Messina, 2022). Growing soybeans to produce casein as well as other non-plant proteins taps into infrastructure that already exists, and therefore is likely to be cost effective. Further, this technology benefits the farming and agricultural communities, by producing value-added crops. This brief narrative review highlights the public health advantages of producing a cheese comprised of soy protein and soybean-derived casein.

## Changes in the type but not amount of animal protein consumed

Perceived barriers that are specific to consuming more plant protein include meat enjoyment and difficulties in preparing vegetarian foods (Fehér et al., 2020) and the belief that meat is essential to a healthy diet (Graça et al., 2019; Valli et al., 2021). Both meat and dairy products play important culinary and cultural roles in society (Sobal, 2005; Kumar et al., 2014; Stoll-Kleemann and Schmidt, 2017; Hargreaves et al., 2021; Nungesser and Winter, 2021). In fact, meat eating has been linked with traditional views of masculinity whereas eating less meat and more plant foods is linked to views of femininity (Rothgerber, 2013; Timeo and Suitner, 2018; Salmen and Dhont, 2023; Stanley et al., 2023), although there is some evidence suggesting these attitudes are changing (Van Der Horst et al., 2023). Consumers associate dairy products with bone health and prevention of osteoporosis (Hagy et al., 2000).

It is notable that although total meat consumption has remained relatively stable over the past 20 years in the US, the type of meat consumed has changed. This shift suggests some willingness among US consumers toward making some dietary changes. Specifically, consumption of beef has declined whereas chicken intake has increased (Kuck and Schnitkey, 2021). Between 1999 and 2019–2020, annual beef consumption declined from 97 pounds per capita to 83 pounds (pork consumption remained relatively stable at about 67 pounds) whereas chicken consumption increased from 89 pounds per capita to 112 pounds (Kuck and Schnitkey, 2021).

Likewise, there has been a marked change in the types of dairy products consumed in the United States. Total milk intake decreased from 0.73 servings/day in 2011–2012 to 0.59 servings/day in 2017–2018 (Tao et al., 2022). This decrease continues a long-term decline in milk intake that began in the 1970s because of changing cultural practices (Stewart et al., 2013, Stewart et al., 2021). This decline occurred long before plant-based milks began to take market share from cow’s milk. Marked declines in cow’s milk intake have also been seen in Germany (Taeger and Thiele, 2024) and other European countries (Autio et al., 2023). Further, there is evidence this trend will continue (Schiano et al., 2022).

However, despite the decline in cow’s milk intake, overall US dairy consumption has increased because of the increase in cheese consumption (Wolf et al., 2020), driven largely by the increased intake of mozzarella cheese on pizza. US per capita intake of non-American type cheese (e.g., not cheddar, Colby or similar cheeses) increased from 6.1 pounds in 1975 to 23.6 pounds in 2022 (2023) U.S. Department of Agriculture and U.S. Department of Health and Human Services, 2020. According to an analysis of the National Health and Nutrition Examination Survey (NHANES) 2017–2020, cheese and pizza are the 5^th^ and 6^th^ largest contributors of saturated fat, respectively, in the US diet (Wambogo et al., 2023). The rise in cheese consumption suggests that producing a cheese containing soy protein and soybean-derived casein that mimics dairy cheese represents potentially fertile ground for increasing plant protein intake.

## Nutritional and functional limitations of existing plant-based cheeses

Plant-based milk alternatives have experienced considerable acceptance among US consumers. Sales of plant-based milks account for 15.3% of total milk sales and over 40% of the US plant foods market whereas plant-based cheese accounted for only 3.8% (Mcclements and Grossmann, 2021) and accounts for less than 1% of all total dollar sales of retail cheese (Short et al., 2021). Short et al. recently noted that the plant-based cheese category is in its infancy compared to plant-based meat and non-cheese dairy alternatives (Short et al., 2021).

Cheese represents a significant obstacle for people interested in consuming more plant protein. Among participants in the UK Biobank study, vegetarians ate almost double the amount of cheese as meat-eaters (Bradbury et al., 2017). Also, a small study of vegetarians found cheese was by far the animal product to which they were most attached (Docherty and Jasper, 2023). To date, both the inferior taste and functional properties of plant-based cheeses have negatively impacted their acceptance by consumers. Yanni et al. (Yanni et al., 2024) have commented that the plant-based cheese industry has not yet been able to replicate the melting and elasticity of cheese and they consider most plant-based cheeses on the market to have a chalky, pasty, and plastic-like texture.

Recently published results of sensory trials in which consumers evaluated 5 different raw plant-based cheeses (100 participants) and 5 different melted cheeses (93 participants) indicated that although the perception was that the cheeses were healthier than dairy cheese, the participants did not like the flavor or textural properties of the plant-based cheeses. (Falkeisen et al., 2022) In agreement, the results of an online survey in Central, Western and Northern European countries, show that for plant-based milks and yogurts, consumers demanded only minor sensory modifications, specifically, towards a less beany and sweet taste. (Waehrens et al., 2023) These products received high liking scores of 7.1 and 7.0 out of 9, respectively. In contrast, lower liking scores were reported for plant-based semi-hard cheeses (5.3 out of 9). The authors of this survey concluded that sensory improvements of plant-based semi-hard cheese alternatives should be directed towards more cheese-like, less artificial and less bland attributes. Finally, a 2023 US consumer survey conducted for the Plant Based Food Association revealed that 73% of plant-based consumers agree with the statement “I wish there was a better plant-based cheese alternative that tasted like regular cheese, melted well, and didn’t have a grainy texture” (Plant Based Food Association, 2023).

The characteristic organoleptic properties of cow’s milk cheese are directly linked to production processes and the different microorganisms used during the fermentation process. According to Masia et al., the colloidal dispersion of fat globules stabilized by casein micelles presents particular behavior upon heat treatment or acidification, and its replication with plant raw materials remains a difficult challenge (Masia et al., 2023). Plant proteins have a higher molecular weight and different functional properties than milk proteins, making it difficult to mimic the texture of cheese. For more information about the role of casein in cheese making please see the reference (Lamichhane et al., 2018).

Many plant-based cheeses are also nutritionally inferior to cow’s milk cheese. Most plant-based cheeses are based on vegetable oils rather than protein. Among the 245 plant-based cheeses in the US market evaluated by Craig et al (Craig et al., 2022), 106 (43%) were based on coconut oil, and another 61 (25%) were comprised of a combination of cashews and coconut oil (**Table 1**). Only 3 of 245 plant-based cheeses provided 10% of the daily value (DV) for protein and 75% had less than 5% of the DV. Also, only 19, 1, and 14 plant-based cheeses were fortified with calcium, vitamin D, and vitamin B12, respectively.

**Table 1:** Composition of US plant-based cheeses*.

	Kcal	Protein (g)	Fat (g)	SFA (g)	Sodium (mg)	Calcium (mg)
All (N=245)	80 (60–100)	0 (0–3)	7 (5–8)	4 (2–5)	190 (150–240	0 (0–0))
Almond (n=7)	70 (60–70)	2 (2–2)	6 (6–6)	0 (0–0)	180 (125–190)	0 (0–0)
Cashew (n=35)	90 (65–120)	3 (2–4)	7 (5–10)	1 (1–2)	130 (98–190)	0 (0–0)
Cashew and coconut oil (n=61)	100 (80–120)	3 (1–3)	8 (7–11)	4 (3–5)	150 (110–200)	0 (0–0)
Coconut oil (n=106)	70 (60–80)	0 (0–0)	6 (5–7)	5 (4–6)	215 (180–258)	0 (0–5)
Oat (n=16)	70 (70–83)	0.4 (0–3)	5 (5–6)	4 (1–5)	200 (200–700)	0 (0–10)
Soy and coconut oil (n=6)	110 (110–110)	1 (1–2)	10 (10–10)	8 (8–8)	205 (190–220)	0-(0–0)
Others (n=14)	80 (80–90)	0 (0–1)	6 (6–7)	3 (2–4)	270 (193–298)	20 (0–20)

Values represent medians (Quartile 1 – Quartile 3).

(Craig et al., 2022).

The findings by Craig et al. (Craig et al., 2022) align with those from surveys of plant-based cheeses in the United Kingdom (Glover et al., 2022), Spain (Fresan and Rippin, 2021) and Greece (Katidi et al., 2023). These surveys highlight the markedly lower protein and calcium content of plant-based cheeses compared to dairy cheeses. In the Greek market, 71% of plant-based cheeses were classified in the Nutri-Score Category E, the lowest score using this scale (A-E) (Katidi et al., 2023). There are notable differences in protein content among the various types of plant-based cheeses as those based on nuts tend to be much higher in protein than those based on oil but are still much lower in protein than dairy cheese.

Among Americans, cheese is the leading source of protein within the dairy group (Pasiakos et al., 2015). Although protein is not considered a short fall nutrient, some US sub-populations may not be meeting protein needs (Weiler et al., 2023; Moughan et al., 2024). Further, some evidence indicates that the recommended dietary allowance is not sufficient for optimal health (Wolfe et al., 2017; Matsumoto et al., 2023). Finally, cheese accounts for 13% of calcium intake of Americans according to an analysis of NHANES 2013–2014, which is second only to milk (19%) (Hoy and Goldman, 2014). Calcium intake of vegans is considerably lower than that of omnivores, a difference that is especially pronounced for females (Bickelmann et al., 2023).

## Producing cheese containing soy protein and soybean-derived casein

Replacing dairy cheese with cheese containing soy protein and soybean-derived casein is one way to increase plant protein intake. However, plant-based cheeses are likely to serve as viable replacements for cow’s milk cheese only with improvements in taste, texture, and functionality. As noted by McClements and Grossmann “The creation of plant-based foods is being held back by a lack of high-quality plant-derived ingredients, particularly proteins, as well as large-scale manufacturing processes to convert these ingredients into desirable end products. In particular, it is still challenging to create analogs of whole muscle meat, fish, yogurt, and cheese because of their complex structural hierarchies” (Mcclements and Grossmann, 2021).

According to Duluins and Baret, strategies to increase consumption of plant protein include 3 narratives: 1) consumer-focused narrative 2) technocentric narrative and 3) socio-technological narrative (Duluins and Baret, 2024). Novel approaches that focus on the production of bioidentical animal protein in plants aligns with the technocentric narrative, which includes development of alternative proteins for food and feed through research, development, technology, and infrastructure. Because of their nutritional value, availability and sustainability, soybeans serve as an ideal plant for these types of processes. As noted previously, soybeans play a major role in the US and world food supply. In the United States, disappearance data indicate that soybean oil alone accounts for 7% of caloric intake (Blasbalg et al., 2011).

Some understanding of the health and environmental advantages of soybeans is relevant to this manuscript because the soybean plant producing casein will also produce soy protein and the cheese made using soybean-derived casein will contain soy protein. Foods made from soybeans have been consumed for centuries throughout much of Asia beginning first in China and then spreading to Japan and other neighboring countries (Guo et al., 2022). Soybeans, like all legumes, fix atmospheric nitrogen due to bacterial symbionts (rhizobia) that inhabit soybean root nodules (Leip et al., 2021), reducing reliance on chemical fertilizer. An estimated half the nitrogen used for crop fertilization globally is lost into the environment, creating environmental concerns (Lassaletta et al., 2014; Melillo, 2021). Soybeans are also widely available as the acreage devoted to soybean production dwarfs that of other dry beans (Hartman et al., 2011). In fact, four times more soybeans are produced globally than all pulses combined. The large number of varieties of this plant allows soybeans to be grown in a wide range of climates and latitudes.

Soybeans also have nutritional advantages over other legumes and plant foods. They are higher in protein (**Table 2**) and the quality of soy protein is similar to animal protein and higher than that of nearly all other plant proteins (Hughes et al., 2011; Rutherfurd et al., 2015; Herreman et al., 2020). Protein quality, which is determined by digestibility and amino acid composition, refers to the ability of a protein to meet the biological requirements for amino acids, and specifically, for the 9 that are indispensable because they cannot be endogenously produced in the quantities needed by the body. In addition to its high quality, soy protein has been investigated for a variety of health benefits, most notably, its ability to directly lower blood cholesterol levels (Blanco Mejia et al., 2019; Jenkins et al., 2019). This attribute of soy protein was formally recognized by the US Food and Drug Administration in 1999 (FDA, 1999). Soy protein also promotes gains in muscle mass and strength in individuals engaged in resistance exercise on par with animal protein including whey (Messina et al., 2018), which is often viewed as the optimal protein for such purposes (Devries and Phillips, 2015).

**Table 2:** Calorie and protein content of selected legumes (raw per 100 g).

Legume	USDA FDC ID	Kcal	Protein	
			g	% kcal
Soybeans	174270	446	36.5	32.7
Lentils	172420	352	24.6	28.0
Pinto beans	175199	347	21.4	24.7
Great Northern	175190	339	21.9	25.8
Kidney beans (red)	173744	337	22.5	26.7
Black beans	173734	341	21.6	25.3
Mung beans	174256	347	23.9	27.6
Peas (green)	170419	81	5.42	26.8
Navy beans	173745	337	22.3	26.5
Adzuki beans	173727	329	19.9	24.2
Lima beans	168396	113	6.84	24.2
Garbanzo beans	173756	378	20.5	21.7

(U.S. Department of Agriculture, Agricultural Research Service, Beltsville Human Nutrition Research Center and FoodData Central., 2019)

By producing plant-grown animal protein, and specifically casein from soybeans, it is possible to produce a cheese that addresses the primary nutritional and functional limitations of existing plant-based cheeses. The generally poor nutritional quality of existing plant-based cheeses, and particularly their low protein content, will be addressed as the new cheese will be based on two concentrated sources of high-quality protein. Functionality will not be a limitation because the cheese will contain casein, the protein in dairy cheese that gives it its unique qualities – such as elasticity and melting.

## Possible benefits of combining soy protein and casein

Combining soy protein and casein into one product may provide a health advantage because the digestion rate of these two proteins differs. Soy protein is digested more quickly than casein, which is slowly digested, likely because gastric acidity causes it to coagulate in the stomach (Mahe et al., 1991, Mahe et al., 1996). Casein digestion may also be impacted by active opioid regulatory peptides, contained in the sequence of some of the casein fractions, and which could play a role by modulating gastrointestinal motility (Daniel et al., 1990). Soy protein is more quickly digested than casein, but more slowly digested than whey protein (Bos et al., 2003; Tang et al., 2009), as the latter is highly soluble in acidic conditions and therefore passes through the stomach and is rapidly hydrolyzed in the duodenum, causing rapid absorption and significant but transient aminoacidemia (Boirie et al., 1997).

Consuming proteins with different digestion rates may favorably impact muscle protein synthesis. Deanne at al. concluded that “Protein blends harnessing the biological benefits of distinct protein sources may represent a means by which to maximise the health benefits of dietary proteins in relation to musculoskeletal health (Deane et al., 2020).” When a slowly digested protein such as casein is ingested, it produces a slower but more prolonged (~6 h) aminoacidemia that results in higher nitrogen retention and less oxidation (Boirie et al., 1997; Dangin et al., 2001) and is effective in stimulating postexercise muscle protein fractional synthetic rate (Dideriksen et al., 2011; Reitelseder et al., 2011). However, because soy protein is more quickly digested than casein, there will be a faster rise in circulating amino acid levels, which may also help to stimulate muscle protein synthesis. Although it remains to be determined whether a soy-casein blend will affect muscle protein synthesis differently than either protein alone, some existing work with protein blends suggests this will be the case (Reidy et al., 2013, Reidy et al., 2014; Borack et al., 2016).

## Possible headwinds against cheese containing soy protein and soybean-derived casein

In recent years, there has been increased focus on the impact of processing on the nutritional and health attributes of food, largely because of the increasing acceptance of the Nova food classification system, which was created by Brazilian researchers in 2009 (Monteiro et al., 2016). Nova classifies all foods into 1 of 4 categories based entirely on the degree to which they have been processed, with the most processed foods belonging to group 4 or the ultra-processed food (UPF) group. Interest in Nova is directly relevant to the development of cheese containing soy protein and soybean-derived casein because regardless of the overall nutritional quality of a food, if it contains concentrated sources of protein, be it whey, casein, soy, wheat, or pea protein, it falls into the ultra-processed category.

Those aligned with Nova discourage the consumption of UPFs and encourage the consumption of group 1, or unprocessed/minimally processed foods. Numerous observational studies have reported positive associations between the intake of UPFs and a range of adverse health outcomes (Barbaresko et al., 2024; Wang et al., 2024). The extent to which these associations are due primarily to differences in diet quality is unclear. Furthermore, in many observational studies, some categories of ultra-processed foods are either not associated with adverse effects or associated with beneficial effects (Monge et al., 2021; Lo et al., 2022; Canhada et al., 2023; Chen et al., 2023; Cordova et al., 2023; Samuthpongtorn et al., 2023; Cho et al., 2024; Li et al., 2024)

In addition, there is clinical research showing that consumption of plant-based meat alternatives, despite being classified as UPFs, leads to several health benefits (Crimarco et al., 2022; Roberts et al., 2022). Finally, a recent analysis in which soymilk (group 4) was compared with cow’s milk (group 1) and soy burgers (group 4) with ground beef (group 1), showed that the soy products do not possess any of the undesirable attributes associated with UPFs more so than their dairy and meat-based counterparts (Messina et al., 2023). The attributes considered were energy density, palatability, eating rate, energy intake rate, satiety, cost, snackability/convenience, and glycemic index. Whether Nova will continue to influence nutrition thinking in the years to come is unknowable, but the existing evidence shows that not all UPFs similarly affect health.

## Summary and conclusions

In recent years a consensus has emerged that for an assortment of reasons, populations of developed countries need to increase their intake of plant protein. Although recommendations vary, moving from a dietary animal to plant protein ratio of 2:1, which is typical in developed countries, to a 1:1 ratio, is a reasonable goal. However, achieving this goal may require the creation of novel foods because consumers have a difficult time making dietary changes.

The introduction of genes into soybeans that code for casein is one way to create novel foods that allow consumers to increase plant protein intake without compromising on taste, functionality, or nutrition. The cheese described in this review, which is based on soy protein and soybean-derived casein, fills a void in the marketplace because plant-based cheeses have not made the inroads into the mainstream that plant-based milks have made. Several reasons have contributed to the relatively weak acceptance of plant-based cheeses.

One is that orosensory properties of plant-based cheeses do not compare well to dairy cheese whereas plant-based milks and cow’s milk are much more comparable. Another is that surveys indicate that consumers of dairy find it more difficult to reduce their intake of cheese than their intake of cow’s milk. For plant-based cheeses to appeal to a broader audience, significant improvements in the quality of plant-based cheeses are needed. Producing casein in soybeans is one way to achieve this goal. Quality in this context refers to functional (melting, stretching, free-oil formation, elasticity and browning), orosensory, and nutritional properties.

Existing plant-based cheeses suffer nutritionally in comparison to cheese produced from cow’s milk. In addition to protein, dairy cheese is a significant source of several nutrients, including calcium, riboflavin, and vitamin B12. Producing cheese containing both soy protein and soybean-derived casein will address the protein deficit. Ideally, these cheeses will be fortified with calcium, riboflavin, and vitamin B12, and perhaps also vitamin D, as these nutrients can be inadequately provided by plant-based diets. Although cost is a major driver of food purchasing decisions, if a cheese containing soy protein and soybean-derived casein can be produced that tastes and functions like dairy cheese and is nutritionally comparable, there is ample reason to think this product will be embraced by consumers who are interested in increasing their intake of plant protein but have not yet been successful at doing so.

Finally, for cheese containing soybean-derived casein to be available in adequate supply, enough soybeans encoded with genes that produce casein need to be grown. Because this soybean offers soybean farmers the opportunity to grow a value-added crop, expectations are that it will be welcomed by the agricultural community. Thus, there are benefits to both the consumer and farmer.

## Author contributions

MM: Conceptualization, Writing – original draft, Writing – review & editing. VM: Writing – review & editing.
